# Effect of Non-Steroidal Anti-Inflammatory Drugs on Sport Performance Indices in Healthy People: a Meta-Analysis of Randomized Controlled Trials

**DOI:** 10.1186/s40798-020-00247-w

**Published:** 2020-04-28

**Authors:** Catherine Cornu, Clémence Grange, Amanda Regalin, Justine Munier, Sonia Ounissi, Natane Reynaud, Behrouz Kassai-Koupai, Pierre Sallet, Patrice Nony

**Affiliations:** 1 INSERM, CIC1407, 69500 Bron, France; 2 UMR 5558, Université Claude Bernard Lyon 1, 69100 Lyon, France; 3 Service de Pharmaco-Toxicologie, Hospices Civils de Lyon, 69000 Lyon, France; 4 Centre d’Investigation Clinique - Hôpital Louis Pradel, 28, Avenue du Doyen Lépine, 69500 Bron, France; 5 Université Claude Bernard Lyon 1, 69100 Lyon, France; 6 ASSOCIATION AFT (Athletes For Transparency), 69100 Lyon, France

**Keywords:** Anti-inflammatory agents, Non-steroidal, Athletic performance, Healthy volunteers, Doping in sport

## Abstract

**Background:**

Non-steroidal anti-inflammatory drugs (NSAIDs) are medications that are frequently used by athletes. There may also be some abuse of these substances, although it is unclear whether NSAIDs in fact enhance performance.

We performed a systematic review and meta-analysis to evaluate the effect of NSAIDs on sport performance indices.

**Methods:**

We selected randomized trials from the PubMed and Cochrane Library databases investigating the effects of NSAIDs on sport performance. Volunteers could be healthy adult men and women. Any NSAID, administered by any route, taken prior to any type of exercise, and for any duration could be used. The control intervention could be a placebo, an active substance, or no intervention. We included double-blind, single-blind, and open-label studies. The primary outcome was the maximum performance in exercises as defined in each study. The secondary outcomes were the time until self-reported exhaustion and the self-reported pain.

**Results:**

Among 1631 records, we retained thirteen parallel-group and ten crossover studies, totaling 366 and 148 subjects, respectively. They were disparate regarding treatments, dose and duration, and the type of exercise. There was neither significant difference in the maximum performance between NSAIDs and control groups nor in the time until exhaustion nor in self-perceived pain.

**Conclusions:**

The existence of an ergogenic effect of NSAIDs on sport performance indices was unable to be concluded, since the level of evidence of the studies is low, the doses tested, and the exercises performed are very heterogeneous and far from those observed in real-life practices. More studies are required.

## Key Points

- Meta-analysis of the effects of non-steroidal anti-inflammatory drugs (NSAIDs) on sports performance indices in healthy individuals showed that the level of evidence in available studies is low, the doses of NSAIDs used are heterogeneous and very different from those administered in ordinary usage, and that the type of exercises performed is very disparate.
- As a consequence, the meta-analysis did not allow conclusions to be drawn about the existence of an ergogenic effect of NSAIDs on sport performance indices.

## Background

Non-steroidal anti-inflammatory drugs (NSAIDs) are a heterogeneous class of drugs chemically unrelated and known to have potent anti-inflammatory, analgesic, antipyretic, and antithrombotic effects. NSAIDs are also associated with an increased risk of adverse gastrointestinal, renal, and cardiovascular effects [1]. In sports medicine, NSAIDs are delivered as oral, topical, intramuscular, or, less commonly, intravenous preparations for treating soft-tissue disorders, joint injury, osteoarthritis, inflammatory arthropathies, fractures, hematoma, and also postoperatively [2, 3].

Athletes use NSAIDs more than any other medication. For example, during the 2000 Olympic Games in Sydney, one in four athletes reported using NSAIDs 3 days before drug testing [4, 5]. During the 2002, 2006, 2010, and 2014 Fédération Internationale de Football Association (FIFA) World Cups, the mean intake was of 0.77 drugs per player and per match; NSAIDs were the most frequently prescribed drugs (36% of drugs), and a similar level of intake was found during the 2003 and 2007 Women’s World Cups [6]. Athletes take NSAIDs for preventing pain, continuing athletic activities in spite of injuries, or accelerate return to function after injury [3, 7, 8]. If taken immediately before or following injury, NSAIDs can reduce musculoskeletal pain and accelerate the return of function [8]; the randomized controlled trial published by Ekman et al. showed that NSAIDs enabled more patients to resume normal walking on days 4 and 7 than placebo or tramadol [8, 9]. However, NSAIDs have side-effects including asthma exacerbation; gastrointestinal and renal side-effects including acute kidney injury; hypertension; and other cardiovascular diseases [3, 10–14]. Therefore, NSAID use presents a potential health risk for athletes.

The World Anti-Doping Agency (WADA) Prohibited List includes any substance and methods that satisfy two of the following criteria: a drug that “has the potential or enhances sport performance,” represents an actual or potential health risk for athletes or violates the spirit of sport described in the WADA Code [15]. NSAIDs are not on the WADA list although they represent an actual or potential health risk for athletes because they are not considered as performance-enhancing drugs [16].

Little is known about NSAID effects on exercise-related physiology and performance. No ergogenic effect might be expected from two studies using them [17, 18]. The action of NSAIDs on pain might enable performing exercise or continuing exercising instead of taking time off training for healing [4]. However, because NSAIDs exert a pharmacologic action on key physiological systems related to exercise performance, a theoretical rationale exists whereby these drugs could provide a significant ergogenic effect [19].

In order to identify whether NSAIDs “have the potential or enhance sport performance,” we conducted a systematic review and meta-analysis of randomized controlled trials, the reference method to update medical evidence for developing clinical practice guidelines and for designing clinical research [20, 21].

## Methods

### Inclusion and Exclusion Criteria

We selected randomized controlled clinical trials investigating the effects of NSAIDs on performance indices in sport. The inclusion criteria included male and female healthy participants including but not limited to athletes, aged over 18 years. The number of participants was not a selection criterion. Any type of NSAIDs, including aspirin, could be used, administered by any route, and at any dose. NSAIDs had to be taken prior to any type of physical exercise, and exposure could be of any duration. The control intervention could be either a placebo, an active substance, or no intervention. We included double-blind, single-blind, and open-label studies. Exclusion criteria were performance criterion unavailable and non-human studies.

The primary outcome was the maximal exercise performance reached, as defined in each study. Secondary outcomes were the time until self-reported exhaustion and self-reported pain. The measure of physical performance could be either a physiological measure (e.g., VO_2_ max) or a measure of strength, acceleration, etc.

### Search Strategy

Two authors (CG, PN) searched the PubMed (which comprises citations for biomedical literature from MEDLINE, life science journals, and online books) and the Cochrane Library databases up to December 2019 with a combination of Medical Subject Headings (MeSH) or equivalent, or text word terms; search strategies were tailored to individual databases. The full search strategy for the PubMed database is shown in supplementary material, Appendix 1. No restrictions on language or year of publication were applied. ClinicalTrials.gov and Researchgate.net were searched for unpublished and ongoing trials. Reference lists in included (and excluded) studies and reviews were also searched for additional studies. The selection of relevant articles was performed by two authors independently (AR, SO, PN, CC) based on the abstract and title. For articles that could not be excluded with certainty on the basis of the title and the abstract, the full text was analyzed.

### Data Extraction

All relevant data were extracted by two authors independently (AR, SO, PN) and included the first author, year of publication, study design, sample size, population characteristics, type of intervention, measured outcomes, and respective numerical values.

When the numerical values of outcomes for studies were not available or needed clarification (*n* = 4), the authors were contacted by email. No response was received in two cases. For these studies, the corresponding values were extracted from graphs using Engauge Digitizer 3.0.

When measurements were performed at different time points, we used the closest to the known peak plasma concentration of each molecule. Given the expected diversity of measured outcomes across studies, the slowest speed for isokinetic exercises was considered; eccentric movements when available, otherwise concentric or isotonic movements, and for sprint tests, the highest speed was used.

### Quality Assessment

Three authors (CG, SO, CC) independently assessed the risk of bias for each study according to the criteria presented in the Cochrane Handbook for Systematic Reviews of Interventions version 5.1.0 [22]. Each study was analyzed for random sequence generation, allocation concealment, blinding of participants and personnel, blinding of outcome assessment, incomplete outcome data, selective reporting, and other bias; the risk of bias arising from each domain is judged as “low,” “high,” or “unclear.” In case of disagreement, a consensus was sought through a discussion between six authors (CG, SO, JM, AR, CC, PN). Publication bias was assessed using a funnel plot [22].

### Statistical Analysis

The meta-analysis was performed using Review Manager (RevMan) Version 5.3., The Cochrane Collaboration, 2014. The results are expressed as standardized mean differences (SMD) with a corresponding 95% confidence interval (CI). A *p* value < 0.05 was considered statistically significant.

In a crossover design, participants are randomized initially to an intervention or control, and then “crossover” to control or intervention, respectively. There are two options to include crossover trials in a meta-analysis: when only data from the first period is available, crossover trials are handled like parallel-group trials. When data for all periods are available, crossover trials should be analyzed separately. Crossover trials that could not be included in the parallel-group meta-analysis were analyzed separately, as recommended by the Cochrane Collaboration [22]. The SMD and the corresponding standard error (SE (SMD)) were computed using the formula recommended by the Cochrane Handbook for Systematic Reviews of Interventions version 5.1.0 part 3 chapter 16.4.6.2. A correlation coefficient for the calculation of the SE (SMD) of 0.5 was chosen arbitrarily; we performed simulations/sensitivity analysis with three other values: 0, 0.25, and 0.75.

The *I*^2^ test was used to quantify the heterogeneity between studies; we considered that heterogeneity might not be important if *I*^2^ < 50%: in such a situation, a fixed-effects model was used. When *I*^2^ was ≥ 50% for a given outcome, a random-effects model was used.

The heterogeneity was addressed by identifying study(ies) likely to bring heterogeneity; identifying the characteristics of this(ese) study(ies), which may create heterogeneity; identifying study(ies) having the same characteristics, and performing analyses with and without the studies potentially leading to heterogeneity. If the heterogeneity disappeared, we could possibly relate the heterogeneity to this(ese) characteristic(s). A subgroup analysis was performed for parallel-arm studies assessing ibuprofen, the most commonly used NSAID in the included studies.

## Results

**Fig. 1:**
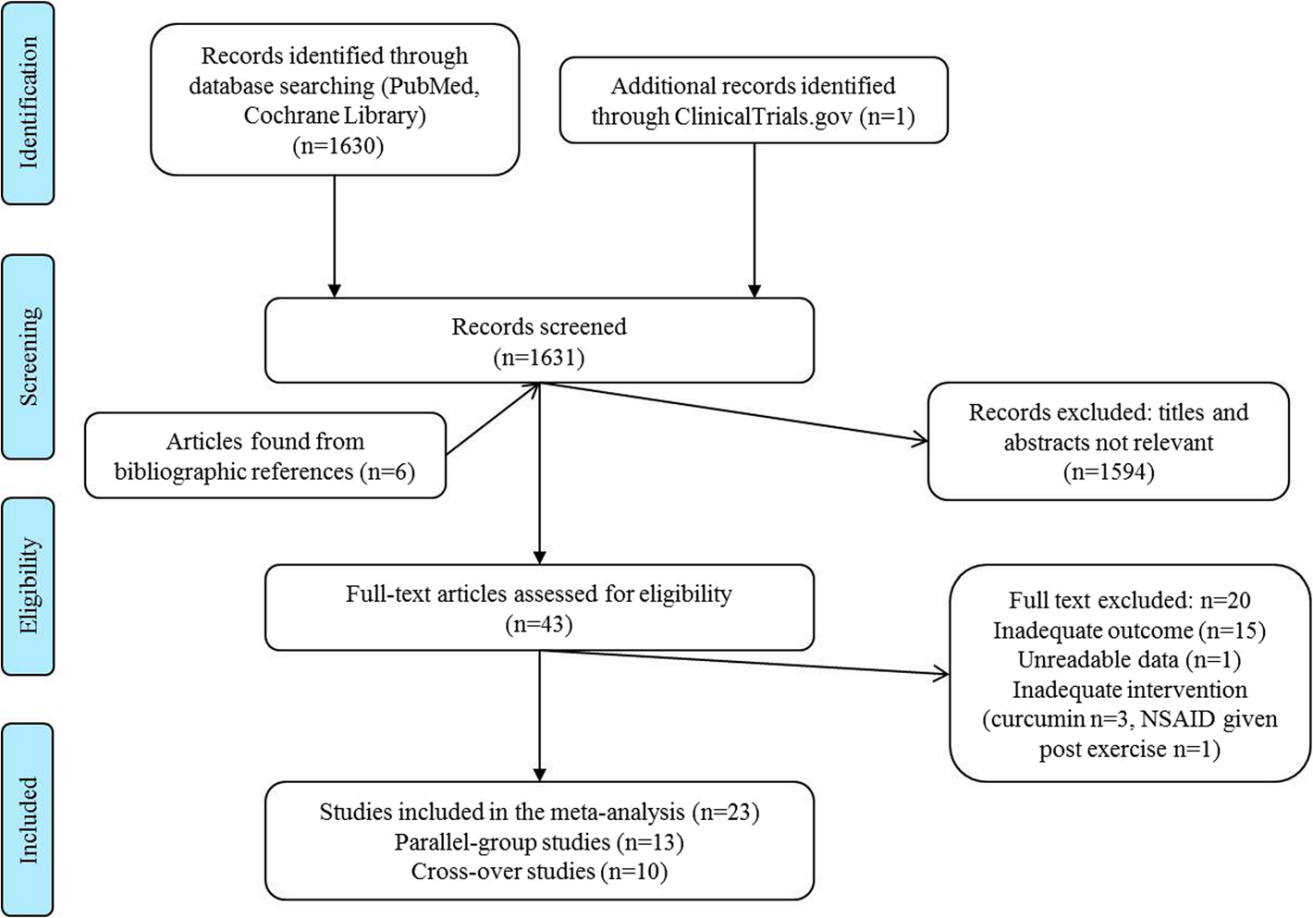
Flow diagram of study selection for the meta-analyses

From a total of 1631 records identified, 23 studies were included in the meta-analysis. A total of 1594 records were excluded based on their title and abstract as they did not evaluate sport performance indices, did not assess the topics of interest, or were animal studies. Forty-nine articles were fully reviewed; from the bibliography of these six, additional articles were selected and reviewed. Twenty articles were excluded because they did not assess the outcomes of interest, and data could not be extracted from the reported graphs, inadequate intervention; a total of 23 studies were included [17, 18, 23–42] (Fig. 1).

The characteristics of the included studies are shown in Table 1. One crossover study could be included in the parallel-group analysis [28]; twelve studies compared ibuprofen to placebo [25–30, 32, 36, 39, 41–43], one study compared ibuprofen to low-dose (75 mg) aspirin [34], one study compared indomethacin to placebo [35] or no treatment [24], three studies compared aspirin to placebo [17, 18, 38], and one study compared flurbiprofen to placebo [40]. One study was conducted in post-menopausal women [29]; other studies included young healthy participants. One study was conducted in an open long-distance setting [27]; all other studies were conducted in a sports science center setting. All studies were randomized controlled trials.

**Table 1:** Characteristics of studies included in the parallel group and in the crossover studies meta-analysis

Author	Study design	Sample size	Population type	Age of participants (in years)	Type of intervention	Type of exercise, measured outcome(s)
Parallel-group design studies						
Donnelly et al. [28]	Double-blind crossover*	32	Men, healthy untrained volunteers	18 to 30	Ibuprofen 1.2 g or placebo 30 min before exercise	Muscle force: isometric contractions Maximum voluntary contraction force (N) Endurance time at 50% of isometric maximal voluntary contraction force (s) at 6 h (first period)—muscle soreness VAS at 6 h
Hasson et al. [30]	Double-blind	10	Men and women, healthy	23.8 ± 4.3	Ibuprofen 400 mg or placebo—4 h before exercise	Muscle force: eccentric contractions Eccentric peak torque (Newton meters) (4 h after drug intake)
Semark et al. [40]	Single-blind	25	Men, active in rugby union and field hockey	19 ± 3	Flurbiprofen 40 mg or placebo patch on each quadriceps—12 h before exercise	Running: 30 m maximal sprints Acceleration during a sprint over 5–10 m (ms^−2^) at 0 h Muscle pain (VAS) at 12 h
VanHeest et al. [42]	Double-blind	24	Men, moderately fit healthy subjects	18 to 34	Ibuprofen 200 mg or placebo 4 times a day 24 h before exercise	Muscle force: eccentric seated knee flexion VO_2_ max during a run on a treadmill (ml kg min^−1^) at 48 h—pain score (VAS) at 24 h
Przybyłowski et al. [38]	Single-blind	19	Men, non-smoking	21 to 23	Aspirin 0.5 g or placebo BID before the exercise and 1 intake 3–4 h before the test	Cycling on a bicycle ergometer until exhaustion (min) Maximal work load (watt) at 3–4 h after last drug intake
Tokmakidis et al. [41]	Double-blind	19	Men and women healthy	24.6 ± 3.9	Ibuprofen 400 mg or placebo one intake just before strength measurement	Muscle force: eccentric contractions Maximal strength 1RM on an eccentric leg curl exercise (kg) Muscle soreness (VAS) at 24 h
Rahnama et al. [39]	Open	22	Men, non-athletic	24.3 ± 2.4	Ibuprofen 400 mg or placebo 1 h before exercise	Muscle force: isometric contractions Maximum eccentric contraction (% of baseline volume) at 24 h
Trappe et al. [43]	Double-Blind	25	Men and women older adults	64 ± 1	Ibuprofen 400 mg TID or placebo	Muscle force: isotonic concentric Increase in muscle strength (kg)
Meamarbashi et al. [35]	Double-blind	27	Men, young healthy non-active students	18.2 ± 0.4	Indomethacin 25 mg or placebo TID during 1 week before exercise	Muscle force: isometric contractions Maximum isometric force (N) at 24 h—muscle pain (Talag Scale) at 24 h
Da Silva et al. [26]	Double-blind	20	Men healthy military long-distance runners	18.8 ± 0.4	Ibuprofen 1.2 g or placebo 1 h before exercise	Muscle force: concentric and eccentric running using a treadmill and a metabolic cart to determine the maximal oxygen uptake (˙VO_2_ max) - Time to exhaustion - Rating of perceived exertion (RPE)
Duff et al. [29], NCT01886196.	Factorial double-blind	90(30 included in meta-analysis)	Women postmenopausal	64.8 ± 4.3	Ibuprofen 400 mg after exercise training only (maximum3 times per week) for 9 months or placebo Resistance training vs flexibility training (placebo exercise)	Muscle force: eccentric and concentric Strength biceps curl (kg)
Lilja et al. [34], NCT02531451.	Single-blind	31	Men and women Healthy	18 ± 35	Ibuprofen 1200 mg control group receiving low dose (75 mg) acetylsalicylic	Muscle force: concentric and eccentric Peak power for the flywheel device
De Souza et al. [27]	Randomized trial parallel groups	20 Primary outcome available for 12 subjects	Men participants in the 42 km Trail Running Challenge	41.1 ± 8.8	Ibuprofen 400 mg or placebo 15 min before the beginning of the race, after 5 h of racing if not yet complete	Long-distance running Counter movement jump performance Finish time (time to exhaustion)
Crossover design studies						
Lisse et al. [17]	Double-blind	17	Men, healthy regular runners	24 to 59	Aspirin 650 mg or placebo 30 min before exercise; wash-out 24 h	Running the subjects’ usual distance 7 times Mean time for a 3.2 km run (min)
Roi et al. [18]	Single-blind	18 Primary outcome available for 9	Men, young athletes	24.7 ± 2.6	Aspirin 1000 mg or placebo 30 min before exercise; wash-out, 1 week	Bicycle exercise Maximum work load (watt) during a at 60 rpm
Lecompte et al. [33]	Double-blind	20	Men, healthy recreationally active	24.0 ± 3.5	Naproxen 500 mg or placebo BID during 1 day before exercise; wash-out, 1 week	Muscle eccentric exercise Muscle strength (eccentric peak torque) 60°/s for the exercise leg (kg m) at day 3
Braun et al. [24]	Open-label	10	Men well-trained distance runners	Unknown	Indomethacin 50 mg PO, or no treatment—TID during 2 days before exercise; wash-out, 2 weeks	Treadmill running VO_2_ (%VO_2_ max) mean data for measurements made after 4, 20, 40, and 59 min of running
Olsen et al. [37]	Open-label	10	Men, healthy	21 to 33 (mean 24)	Indomethacin 100 mg or no treatment PO—10 h just before exercise; wash-out period, 7 days	Bicycle exercise at 60 rev./min Maximum work load (watt) and VO_2_ at 75% of the individual VO_2_ max (mL/min) during an exercise session
Baldwin et al. [23]	Double-blind	15	Men and women, old, healthy	60 ± 2	Naproxen 220 mg or placebo TID for 10 days	Muscle isometric exercise Maximal isometric force Muscle soreness
Hudson et al. [31]	Double-blind	15	Men college-aged	22.0 ± 1.3	Aspirin 10–10.4 mg/kg or placebo 1 h before testing	Muscular concentric and eccentric Perceived exertion Perceived pain index
Krentz et al. [32]	Counter-balanced, double-blind	18	Men and women	24	Ibuprofen 400 mg or placebo immediately after training daily for 5 weeks	Muscular concentric Muscle soreness
Correa et al. [25]	Double-blind	12	Men, regularly trained	22.8 ± 3.2	Ibuprofen 1.2 g or placebo 1 h before training	Muscle concentric and eccentric exercise
Morgan et al. [36], part A	Double-blind	13	Men, physically active	7	Ibuprofen 400 mg (combined with 600 mg maltodextrin) or 1000 mg of maltodextrin (placebo) orally, 134 min prior to the exercise	Muscle force: isokinetic Peak MVC (N m)

*TID* three times a day, *BID* two times a day, *VAS* visual analog scale VO2max, *MVC* maximum voluntary contraction

*Crossover studies for which data were available at the end of the first period were included in the parallel-group meta-analysis

A total of 296 participants were included in the parallel-group primary outcome meta-analysis (12 parallel-group studies and 1 first period of crossover studies) and 135 participants in the crossover meta-analysis (10 crossover studies). One study included post-menopausal women [29], one included older men and women [43], six included both males and females [23, 30, 32, 34, 41, 43], and all other studies included male subjects. Seven parallel-group studies included athletic, well-trained, or young people who regularly practiced sport [17, 18, 24–27, 40, 42]; the other studies included untrained, non-active, or non-athletic subjects. All participants were healthy.

One study investigated a transdermal route for NSAID administration [40]. Seven parallel-group studies and three crossover studies investigated an acute administration whereas other studies investigated a longer period of treatment that ranged from 10 h to 1 week before exercise. The dose of ibuprofen investigated ranged from 400 mg to 1.2 g per intake, aspirin from 500 mg to 1000 mg per intake, and indomethacin from 75 mg to 150 mg per day.

Interventions and outcome measurements varied across trials and are shown in Table 1. Sixteen studies used force exercises: five used isometric force, nine used eccentric force, and two isotonic/isokinetic force; thirteen had force outcome measures: ten used maximal contraction, two used peak torque, and one used peak power. Four studies used running exercises: long-distance running (one study), one used jump performance after running, short distance (one study, with time for running 3.2 km as outcome), and one sprint running (with acceleration as outcome). Three used treadmill running with VO_2_ max as outcome. Three studies used bicycle exercise and measured the maximum workload.

### Quality Assessment

**Fig. 2:**
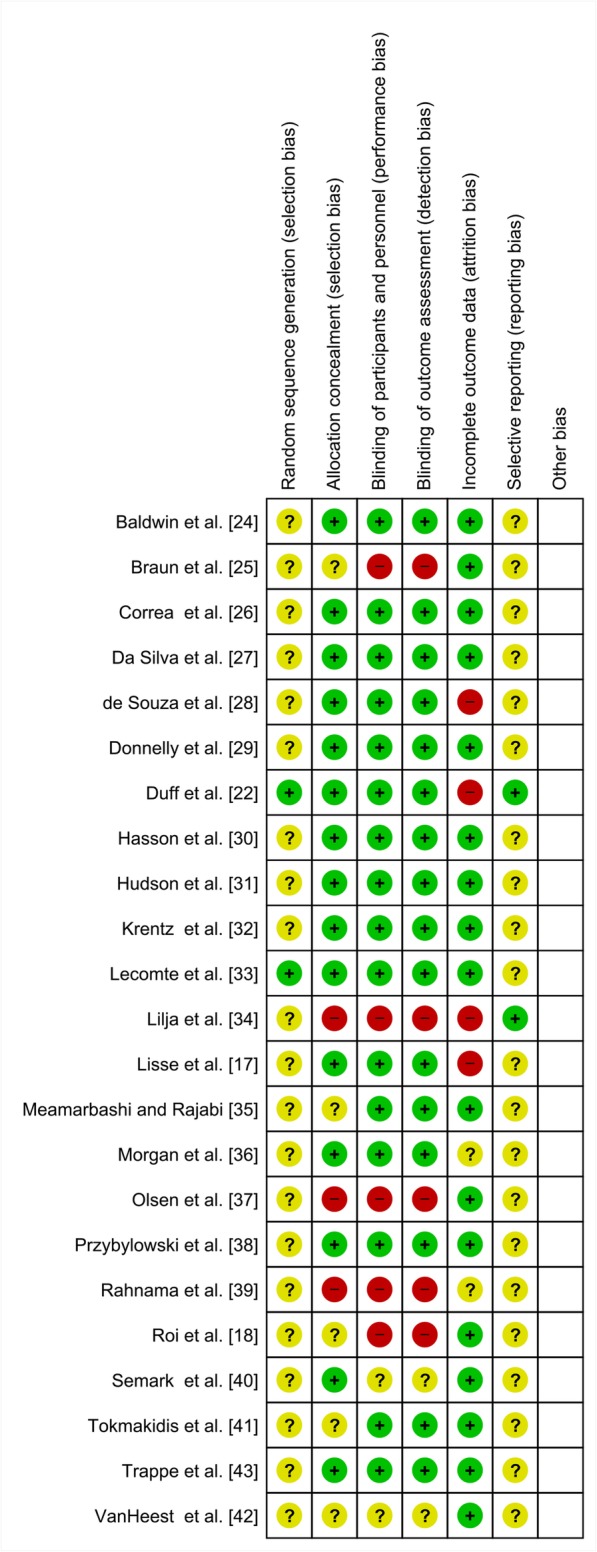
Risk of bias summary: review authors’ judgments about each risk of bias item for each included study. Green stands for low risk, red stands for high risk, and yellow for unclear risk

Quality assessment of the included studies is shown in Fig. 2. One parallel-group study was open-label [39], and three were single-blind studies [18, 34, 40], with a substantial risk of bias. Incomplete outcome data (attrition bias) was present in five studies. Selective reporting bias could be excluded for only three of the included studies since the study protocols were registered. Therefore, in most studies, some planned outcomes may not have been reported and, conversely, unplanned outcomes may have been reported.

### Outcome Measures

#### Primary Outcome: Maximum Performance

##### Parallel-Group Meta-Analysis

**Fig. 3:**
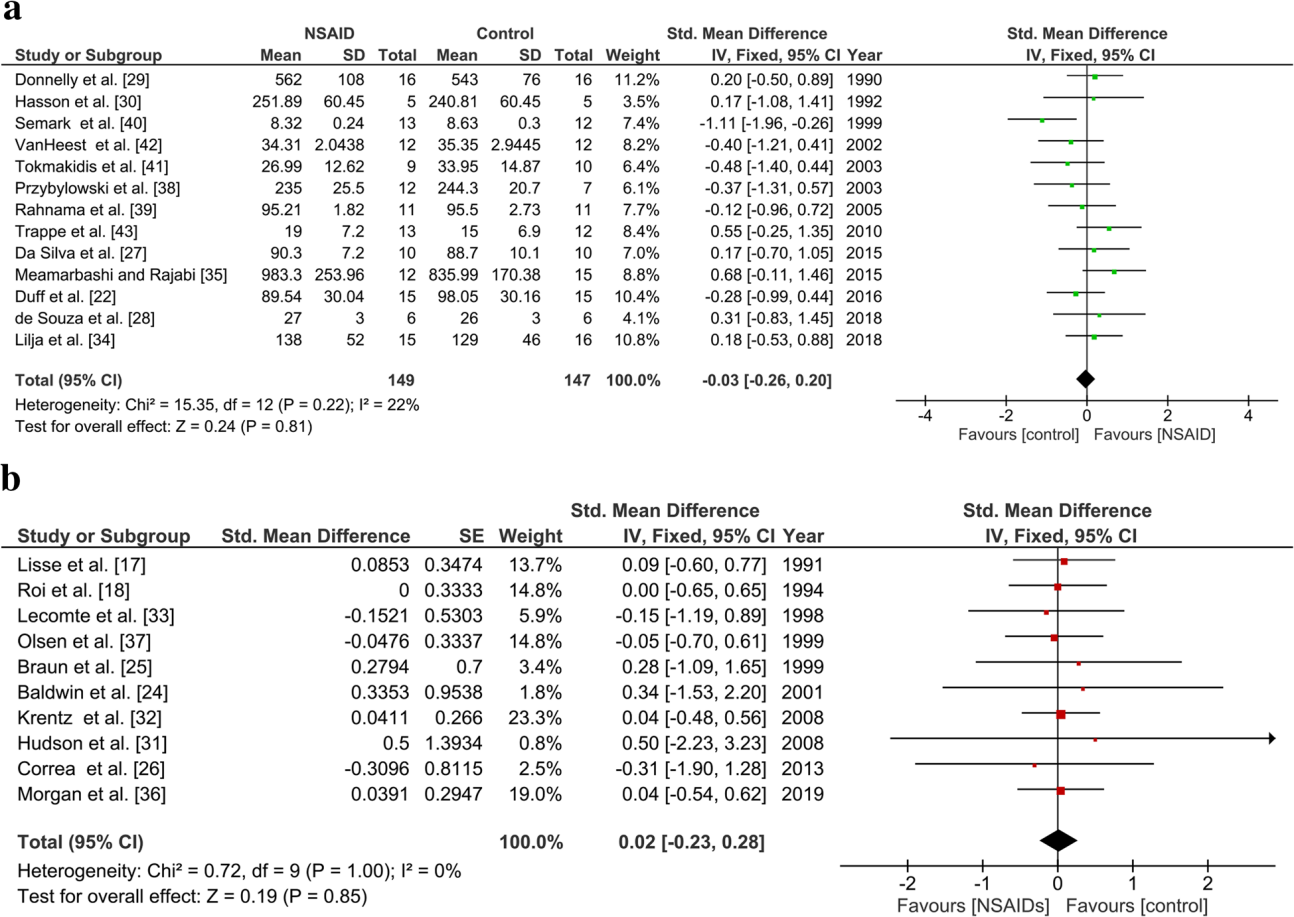
Forest plot of the primary outcome, maximum performance **a** in the parallel-group studies and **b** in the crossover studies (correlation coefficient = 0. 5)

Thirteen studies reported the maximum performance, totaling 149 participants in the NSAID group and 147 participants in the control group. There was no significant difference in the maximum performance between groups (SMD, − 0.03; 95% CI [− 0.26, 0.20]) using a fixed-effects model (*I*^2^ = 22%; Fig. 3a).

##### Crossover Meta-Analysis

Nine studies reported a maximum performance index, totaling 135 participants. There was no significant difference in the maximum performance indices between groups (SMD, 0.01; 95% CI − 0.39, 0.40) using a fixed-effect model (*I*^2^ = 0%) and a correlation coefficient of 0.5 (Fig. 3b). Analyses with different values of the correlation coefficient showed similar results, all being non-significant.

#### Secondary Outcomes: Time Until Exhaustion

##### Parallel-Group Meta-Analysis

**Fig. 4:**
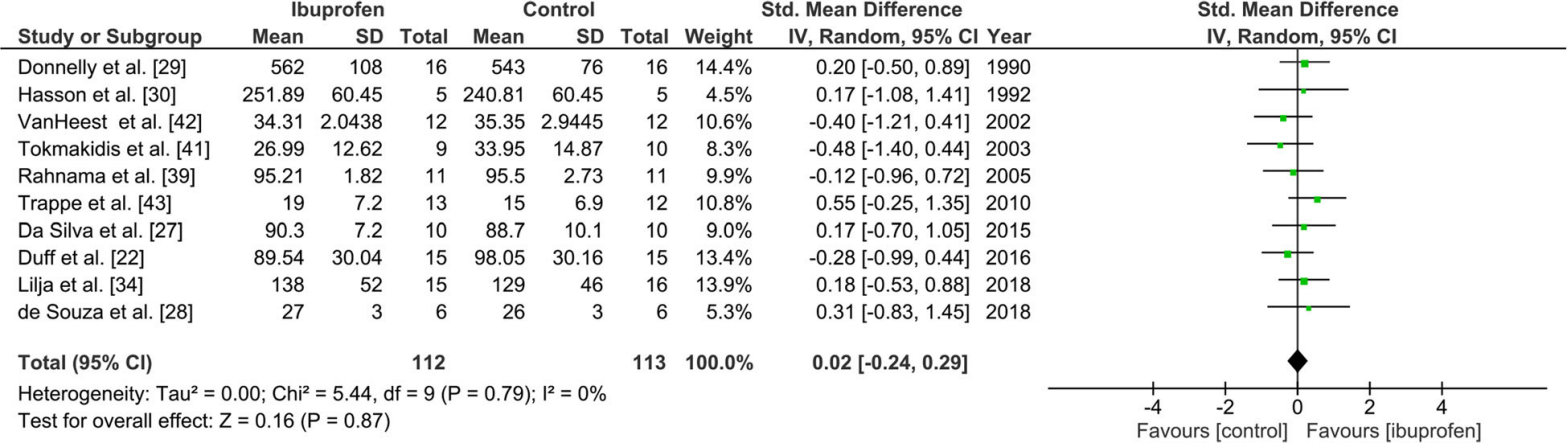
Forest plot of the secondary outcome, time until exhaustion in the parallel-group studies

Four studies reported the outcome “time until exhaustion,” totaling 44 participants in the NSAID group and 39 participants in the control group. There was no significant difference in the time until exhaustion between groups (SMD, − 0.11; 95% CI − 0.55, 0.33) using a fixed-effect model (*I*^2^ = 0%; Fig. 4). Time until exhaustion was not available for crossover studies.

#### Secondary Outcomes: Self-Perceived Pain

**Fig. 5:**
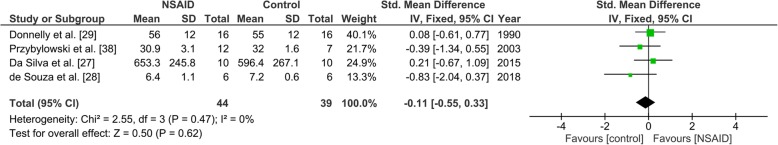
Forest plot of the secondary outcome, self-perceived pain in the parallel-group studies

The assessment of the self-perceived pain was available for six studies, totaling 72 participants in the NSAID group and 75 participants in the control group. Self-perceived pain was not significantly lower in the NSAID group than in the control group (SMD, − 0.32; 95% CI − 0.65, 0.01) using a fixed-effect model (*I*^2^ = 36%; Fig. 5). Self-perceived pain was not available for crossover studies.

#### Sub-Group Analyses: Ibuprofen Versus Control Group

##### Parallel-Group Meta-Analysis

The maximum performance reached by the participants in the trials assessing ibuprofen versus placebo was available for ten studies totaling 112 participants in the ibuprofen group and 113 participants in the control group. There was no significant difference in the maximum performance between groups (SMD, 0.02; 95% CI − 0.24, 0.29) using a fixed-effect model (*I*^2^ = 0%; Supplementary Figure 1).

#### Heterogeneity

There was no heterogeneity in any analysis.

#### Adverse Effects

No adverse effects were reported in the studies included. History of gastrointestinal disorders was an exclusion criterion in all studies.

#### Correlation Coefficient of the Crossover Trials Analysis

The sensitivity analysis performed using 0, 0.25, and 0.75 values for the correlation coefficient found the same results as the analysis using the correlation coefficient of 0.5 (results not shown).

#### Publication Bias

The number of included studies was insufficient to allow a robust interpretation of funnel plots. However, the funnel plot for the primary outcome (Supplementary Figure 2) was well distributed.

## Discussion

Because NSAIDs exert a pharmacologic action on key physiological systems related to exercise performance, a theoretical rationale exists whereby these drugs could provide a significant ergogenic effect [19]. They are widely used for concealing pain and therefore allowing continuing a sports activity.

This meta-analysis did not show that NSAIDs significantly impacted sport performance indices in healthy people. NSAIDs did not reduce time to exhaustion, or self-perceived pain, although there was a trend toward a reduction of pain.

Studies included in this meta-analysis are very disparate. For instance, some studies included athletic, well-trained, or young people who regularly practiced sport; others included untrained, non-active, or non-athletic subjects; and one included post-menopausal women. The treatments investigated differed in terms of drug, dose, and duration of treatment, and finally, the type of exercise also differed between studies. The reported outcomes were different, and this is why we used the standardized mean difference [44] for the measurement of association in the meta-analysis. The assumption behind this use is that the differences in standard deviations among studies reflect differences in measurement scales and not real differences in variability among study populations. This assumption may be problematic when real differences in variability between the participants in different studies are expected. Here we can assume that despite differences between studies, the similarities of their setting, the inclusion of healthy participants, and the use of drugs of a unique class means the use of SMD is not flawed.

All but one study were performed in an experimental setting, such as a sports science center setting, and their designs do not mimic real practices of athletes. In most studies, the drug was administered before exercise, immediately before or up to 12 h before; in two studies, it was administered three times or four times a day from 24 h to 1 week before exercise. The doses used were much lower than the dangerous overuse taken by athletes during competitions, i.e., parenteral administration, concomitant use of several different NSAIDs on the same day, inappropriate dosing (too low or too high dosing, e.g., up to 8 tablets of diclofenac in one day), and drug interactions ( with asthma treatment, with antibiotics); use of outmoded drugs such as phenylbutazone, (used 11 times, 6 by injection), a potent NSAID with a long half-life, has a wide range of adverse effects, particularly relating to the stomach and bone marrow, in even small doses, as underlined by Corrigan and al [4]. The exercise was a single bout exercise and was not as prolonged as in competitions. Therefore, these results could not be extrapolated to sport competition conditions.

Considering NSAID side effects, the study reported by Lipman et al. was aimed at evaluating ibuprofen versus placebo effect on acute kidney injury in ultra-marathons [45]. The conclusion of the authors was that ibuprofen non-significantly increased the incidence of acute kidney injury by 18% (95% CI − 4% to 41%). This study, which was performed in a real setting of an ultra-marathon, did not report performance outcome and could not be included in this meta-analysis. However, Lilja et al. conclude that “maximal over-the-counter doses of ibuprofen attenuate strength and muscle hypertrophic adaptations to 8 weeks of resistance training in young adults” [34]. Thus, young individuals using resistance training to maximize muscle growth or strength should avoid excessive intake of anti-inflammatory drugs. Adverse effects were not addressed in the other studies included in this meta-analysis. As said before, these studies included only healthy subjects and required a very short-term use of NSAIDs; they were therefore not designed to address the question of adverse effects. Although acute kidney injury is a common finding in endurance runners, encountered in 34–85% of ultra-marathoners, and NSAIDs are thought to contribute to acute kidney injury, 35–75% of ultra-marathoners ingest them during competition [45]. In addition, a double-blind randomized placebo-controlled trial performed under marathon conditions showed increased rates of acute kidney injury in those who took ibuprofen, and although not statistically inferior to placebo by a small margin, there was a number needed to harm of 5.5 people to cause 1 case of acute kidney injury [45]. A similar design could be used to evaluate the ability of NSAIDs to enhance sport performance. The study should be a randomized, double-blind, placebo-controlled clinical trial with time until exhaustion during a running exercise or the performance during a time-trial, a semi-marathon, or a marathon as a primary outcome.

This meta-analysis has some limitations. The search was performed in a limited number of databases; however, it has been shown that using data sources beyond PubMed has a modest impact on the results of systematic reviews of therapeutic interventions [46]. The methodological quality of studies included is poor, the risk of bias is high, and a publication bias cannot be excluded. The funnel plots is symmetric, but the number of studies for each outcome is low, and most of these studies were not registered before being conducted as recommended for comparative studies [47]. Thus, the findings of this work indicate that there is a lack of studies in this area.

## Conclusion

Our meta-analysis does not allow concluding unambiguously on the existence or not of an ergogenic effect of NSAIDs on sport performance. The risks for athletes are not well documented, and there is still a lack of studies adequately designed in this area. The absence of evidence is not evidence of the absence of an effect; since this meta-analysis did not show any effect on pain, this suggests that the trials were not optimally designed. We recommend the conduct of new trials, adequately powered, methodologically sound, using adequate dosage in a real-life setting to address the question of whether NSAIDs have an influence on athletic performance, and provide data for adequate classification by WADA.

## Supplementary information

**Additional file 1: Supplementary Figure 1.** Forest plot for the primary outcome, maximum performance in the subgroup of trials assessing Ibuprofen

**Additional file 2: Supplementary Figure 2.** Funnel plot for the primary outcome, maximum performance

## Data Availability

No additional data available. Only published data were used.

## Abbreviations

CI: Confidence interval
FIFA: Fédération Internationale de Football Association
MeSH: Medical Subject Headings
NSAIDs: Non-steroidal anti-inflammatory drugs
SE: Standard error
SMD: Standardized mean differences
WADA: World Anti-Doping Agency
