# Supplementary figures and images for: Effect of Non-Steroidal Anti-Inflammatory Drugs on Sport Performance Indices in Healthy People: a Meta-Analysis of Randomized Controlled Trials
Source: Sports Med Open. 2020 Apr 28;6:20. doi: 10.1186/s40798-020-00247-w (PMC7188752; doi:10.1186/s40798-020-00247-w)

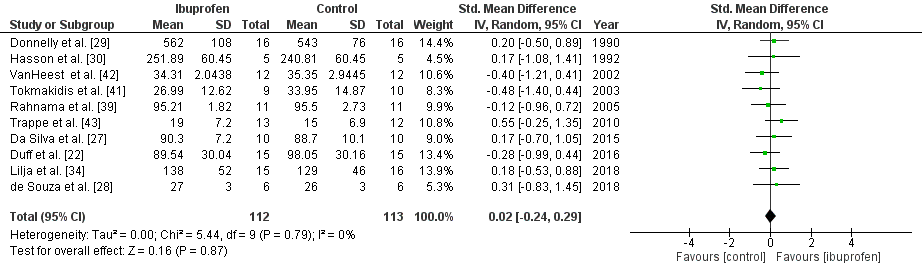

Supplement: Supplementary file 1 — Additional file 1: Supplementary Figure 1. Forest plot for the primary outcome, maximum performance in the subgroup of trials assessing Ibuprofen [file 40798_2020_247_MOESM1_ESM.png]

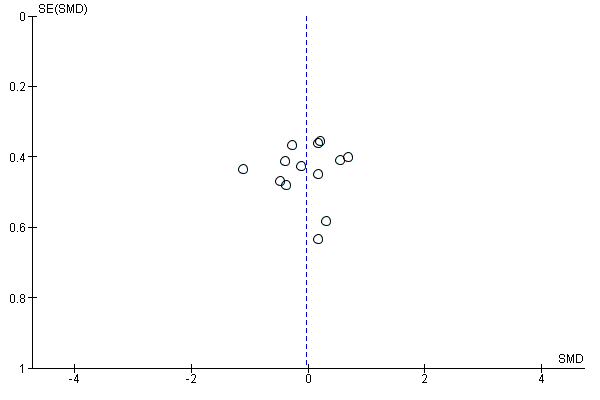

Supplement: Supplementary file 2 — Additional file 2: Supplementary Figure 2. Funnel plot for the primary outcome, maximum performance [file 40798_2020_247_MOESM2_ESM.png]
